# Corpus Refactoring: a Feasibility Study

**DOI:** 10.1186/1747-5333-2-4

**Published:** 2007-09-13

**Authors:** Helen L Johnson, William A Baumgartner, Martin Krallinger, K Bretonnel Cohen, Lawrence Hunter

**Affiliations:** 1 Center for Computational Pharmacology, University of Colorado School of Medicine, Aurora, CO, USA; 2 Structural Computational Biology Group, Spanish National Cancer Research Centre, Madrid, Spain

## Abstract

**Background:**

Most biomedical corpora have not been used outside of the lab that created them, despite the fact that the availability of the gold-standard evaluation data that they provide is one of the rate-limiting factors for the progress of biomedical text mining. Data suggest that one major factor affecting the use of a corpus outside of its home laboratory is the format in which it is distributed. This paper tests the hypothesis that *corpus refactoring *– changing the format of a corpus without altering its semantics – is a feasible goal, namely that it can be accomplished with a semi-automatable process and in a time-effcient way. We used simple text processing methods and limited human validation to convert the Protein Design Group corpus into two new formats: WordFreak and embedded XML. We tracked the total time expended and the success rates of the automated steps.

**Results:**

The refactored corpus is available for download at the BioNLP SourceForge website http://bionlp.sourceforge.net. The total time expended was just over three person-weeks, consisting of about 102 hours of programming time (much of which is one-time development cost) and 20 hours of manual validation of automatic outputs. Additionally, the steps required to refactor any corpus are presented.

**Conclusion:**

We conclude that refactoring of publicly available corpora is a technically and economically feasible method for increasing the usage of data already available for evaluating biomedical language processing systems.

## Background

Biomedical corpora are essential for the development and evaluation of biomedical language processing (BLP) tools. For instance, Tsuruoka et al. [1] show that their biomedical POS and named entity taggers perform better when trained on biomedical corpora instead of the Wall Street Journal corpus. Also, the availability of annotated corpora in standardized formats is essential to compare different BLP tools against each other [2].

Cohen et al. [3] surveyed the usage rates of a number of biomedical corpora, and found that a small subset of them represented the majority of uses of these publicly available data sets: most biomedical corpora have not been used outside of the lab that first created them. It is not known how many person-hours went into the construction of these resources, but it is likely that they represent many person-years and hundreds of thousands of dollars, not to mention the domain expertise – a considerable investment of human and capital resources. Most corpora remain unused, despite the fact that availability of the sort of gold-standard evaluation data that they provide is arguably the rate-limiting step in the progress of biomedical text mining.

Empirical data on corpus design and usage presented in Cohen et al. [3,4] suggest that one major factor affecting the use of a corpus outside of the laboratory in which it was produced is the format in which it is distributed. Although there is no universally accepted standard for corpus encodings, the distribution in some well-known format seems to be a prerequisite for acceptance of a corpus by the community at large [5-7]. A number of corpora containing high-quality semantic information languish unused today, largely due to idiosyncratic formatting of their contents and/or lack of annotation into the text. Smith et al. [7] showed that when corpora use similar and relatively standard embedded annotation formats, standardizing their formats (and their semantics) was both practical and valuable.

These findings suggest that there would be a large benefit to the community in refactoring these corpora. *Refactoring *is defined in the software engineering community as altering the internal structure of code without altering its external behavior [8]. In the context of corpus linguistics, we refer to refactoring as changing the *format *of a corpus without altering its *contents*, i.e. the annotations, the metadata, and the text that those describe. The significance of being able to refactor a large number of corpora should be self-evident: a likely increase in the use of the already extant publicly available data for evaluating biomedical language processing systems, without the attendant cost of repeating their annotation. But, how feasible would corpus refactoring be? How much of the process is automatable? How many person-hours would be required to repair errors in the automated outputs? This paper examines those questions directly.

We examined the question of whether refactoring corpora is practical by attempting a proof-of-concept application: modifying the format of the Protein Design Group (PDG) corpus [9]. This work contrasts with the work by Smith et al. [7] in that the PDG corpus is a *metadata corpus *and not a corpus in a standard or embedded format. We use the term metadata corpus to mean a collection of texts that, unlike a *document collection*, lists information related to specific substrings in the text, but that unlike the typical *annotated corpus*, encode this information without any indication of the location of those substrings within the text itself, (see [10] for a complete description).

We refactored the PDG corpus from its current idiosyncratic format to a stand-off annotation format, WordFreak [11,12], and a format similar to the Genia Project Markup Language (GPML) [13] embedded XML format. To do this, we performed a semi-automatic modification of the format, using simple text processing to perform most of the work and relying on manual intervention only to validate the transformation and to handle cases that could not be processed automatically.

To evaluate the feasibility hypothesis, we examined all outputs at every step of the refactoring process. We quantified errors made by the automatic portion of the work flow and the time spent manually validating data and correcting errors. The resulting output – the Protein Interaction Corpus (PICorpus) – is freely available at the BioNLP SourceForge website [14]. This work demonstrates that corpus refactoring is largely automatable, that it can be achieved at low cost, and that it results in useful and usable outputs.

## Methods

### The PDG Corpus

**Figure 1:**
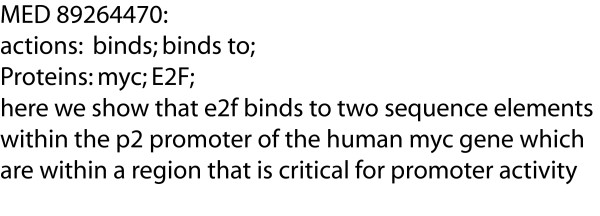
**Text block from original PDG corpus**. This block of text from the original PDG corpus shows the idiosyncratic format of the protein interaction annotations. "MED" is a deprecated MEDLINE ID. The words that follow "actions" are keywords denoting an interaction type between proteins. The words that follow "Proteins" are the interactors. The text that follows has been altered from the original MEDLINE publication.

The original PDG corpus was constructed by automatically detecting protein-protein interactions of two signalling pathways in Drosophila, using the system described in Blaschke et al. [9], and then manually reviewing the output. Within the corpus, the data is distributed across two sections in two different formats, corresponding to the two Drosophila systems. The section of the original corpus used in the refactoring process is the second one, and it is that subset of the entire corpus that we mean when we refer to the PDG corpus from now on. It is composed of blocks of unannotated text and meta-information that describe the protein-protein interactions mentioned in text, (see Figure 1). Each block of text has the following characteristics:

• a MEDLINE ID

• a list of interaction types, separated by semicolons

• a list of proteins, separated by semicolons

• a string of text in which the interactions and protein interactors are mentioned

This data comprises a small corpus of 10,291 words, about 283 sentences, and 417 protein-protein interactions. It contains some residual errors in entity identification and in relation extraction from the automatic processing steps, which are described in the Results section. This data could potentially serve as evaluation data for systems that extract information on protein-protein interactions, an important factor in human disease [15], as well as for other tasks, such as entity identification.

As Cohen et al. [4] point out, the PDG corpus was built at the very beginning of the involvement of the computational biology community in text mining efforts. Its eventual public distribution was not anticipated at the time of its construction. For these reasons, it is understandable that the data was not prepared in any typical corpus format. The data was originally made available in an HTML file, which necessitated stripping HTML tags before even such simple tasks as performing a word count on the corpus could be carried out.

We selected the PDG corpus for our pilot project for several reasons. The PDG corpus is the smallest publicly available biomedical corpus of which we are aware, which suggested that manual validation times were more likely to be within reasonable bounds than for any other corpus. In other ways, the PDG corpus presents considerable challenges that refactoring other corpora would not. First of all, the format of the data is completely idiosyncratic – no other corpus is encoded quite like it. The process of refactoring the PDG will bring to light how to best handle other idiosyncratically encoded corpora, of which there are many.

Secondly, as noted, the PDG is a metadata corpus, meaning there are no mark-ups mapped to specific strings in the text. This introduces a number of challenges in mapping the original metadata to actual annotations that some biomedical corpora would not present, since many biomedical corpora contain annotations in the strict sense of that word. Therefore, the PDG corpus, with its idiosyncratic format and lack of annotation, provided an unusually stringent test of the feasibility hypothesis.

### Refactoring methods

The structure and contents of the original corpus suggest the logical steps of the refactoring process, listed here and explained in detail below.

1. Retrieve the original abstract.

2. Locate the original source sentence in the title or abstract.

3. Locate the interaction type keywords and the entities (i.e., proteins) in the text.

4. Produce output in the new formats.

A variety of facts about the nature of the original corpus posed challenges for the refactoring. First, the MEDLINE IDs used in this corpus have since become deprecated. Also, the text in the original version of the corpus was altered from the MEDLINE records in a number of ways, described in step 2 below. Finally, protein names in the metadata were often altered by case toggling and removal of punctuation.

Various obstacles had to be overcome at each of these steps. Steps 1–3 required manual validation of the outputs, and sometimes manual correction, as well. We wrote code to automatically process various aspects of the steps. A human curator then manually examined the output of each step, correcting it where necessary so that the output at each stage was completely correct. In a few situations, curators changed the content of the corpus. While this calls into question whether what we did constitutes corpus refactoring as we have defined that term, we found it necessary in a few specific instances, detailed in steps 2 and 3 below and discussed in the Conclusion section.

**1. **To recover the original text from PubMed, the first step was to look up the PubMed ID of each of the corpus blocks. The PDG corpus references each evidence of protein interaction by a MEDLINE ID number. These are deprecated identifiers, so we mapped them to PubMed IDs by submiting a query to the NCBI eutils MEDLINE UI/PubMed ID matcher web site [16] which returned the PubMed ID. We then used the PubMed IDs to retrieve the corresponding abstracts automatically.

To validate the MEDLINE-to-PubMed mapping, we simply verified the presence of a PubMed ID in the output. When a null ID was detected, the curator manually retrieved the PubMed ID and abstract by searching PubMed with the text provided in the original corpus.

**2. **The next step in the mapping process was to find the raw text in the PubMed abstract. Sentences in the original PDG corpus have been altered by clause tokenization, case folding, and punctuation removal. This made it impossible to rely on string-matching to recover the original sentences. Instead, we started by segmenting the sentences of the abstract [17]. The sentence from the abstract with the highest Dice coefficient as compared to the original corpus text was chosen.

To verify that the correct sentence had been extracted, curators accessed a file that had, among other information, the full text of the retrieved abstract, the original sentence, and the sentence our system extracted. If the sentences matched, the curator did nothing. If the sentences did not match, the curator read the abstract to find the correct sentence(s). The curator copied the correct text in place of the incorrectly or incompletely extracted sentence. In cases where there was no match between abstract sentences and original text, the curator searched PubMed for the title and abstract of the publication by entering the PubMed ID and choosing the appropriate text from PubMed. In cases where the original corpus text did not span an entire sentence, the automatic sentence extractor expanded the text span to the sentence boundaries, and the curator verified the expansion. This was done to preserve the context around the protein interaction concepts in text.

**3. **The third step in the corpus construction process was to transform the interaction type and protein metadata into annotations in the text. The interaction keywords appear in the original metadata in the same form as they do in the text (with the exception of case-toggling), but many of the proteins have been altered in terms of case, whitespace, digits and punctuation. Again, this made string-matching unreliable. Our search algorithm automatically constructed regular expressions to find the text spans of proteins and interaction keywords, allowing for optional punctuation, and permitting optional hyphenation or whitespace before and after digits in protein names. The search algorithm then determined zero-based offset values for beginning and end characters of interaction words and proteins.

To validate the mapping from metadata to text, the curators were presented with files that contained the extracted sentence with tags around the annotated interaction keywords and proteins. If the span of the character offsets in the automatic output was incorrect, the curator fixed the offset span. If the entity matched was not a valid interaction word or protein, the curator removed the entity from the metadata list. In some cases, the automatic entity matcher did not find the entity in the extracted sentence, in which case the curator either added the offset values based on the correct entity in the sentence, or removed the entity from the metadata if there was no valid corresponding entity in the sentence.

**4. **Finally, the curated data from the last step was programatically converted into the WordFreak and embedded-XML formats via an application centered around the Unstructured Information Management Architecture (UIMA) [18-20]. Sentences and the associated annotation data were imported into the UIMA framework where they were placed in a standardized data structure, and then outputted in their refactored form by an output-printer component. Separate output-printers were developed for the WordFreak and embedded-XML output formats, both of which are completely reusable for future refactoring efforts. The design of the application and incorporation into the UIMA architecture promotes system extensibility. A new output-printer can be developed and plugged into the system without altering any upstream components. Similarly, a different corpus, once placed in the UIMA standardized data structure, can be refactored using any of the available output-printers.

Validation of the final output-production step consisted of checking for file format validity.

### Output Formats

We produced our refactored corpus in two formats. One is the WordFreak format used by the PennBioIE corpus [11,12]. WordFreak presents annotated corpus data in a stand-off format, meaning that the annotation information is in one file and the raw corpus text is in a separate file. Annotation tags are represented apart from the original text as sequential values that refer to the beginning and end character position of the entity in the corpus text file. The other format is embedded XML similar to the GENIA project's GPML mark-up language [13]. This format has the disadvantage that the annotation process alters the original text by adding the annotation tags in-line. On the other hand, the advantage is that there is no mapping back from character offset values to the raw text.

We selected these formats for two reasons. One was that, according to a corpus usage survey, there is evidence suggesting that stand-off annotation and embedded XML are the two most highly preferred corpus annotation formats in the BLP community [21]. In fact, 50% of the survey respondents said they preferred the embedded XML format. Another was that these two formats are employed by the two largest extant curated biomedical corpora, and there may be value in a move towards format standardization.

It is important to note that, while we settled on these output formats for this project, virtually any annotation format may be rendered using the process described in this project with little further effort or expense.

## Results and Discussion

### Format of the refactored corpus

**Figure 2:**
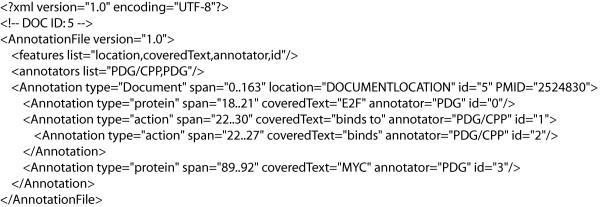
**Refactored corpus: Word Freak format**. Example of the text block from Figure 1 in the refactored WordFreak format. The original sentence reads *Here we show that E2F binds to two sequence elements within the P2 promoter of the human MYC gene which are within a region that is critical for promoter activity*.

**Figure 3:**
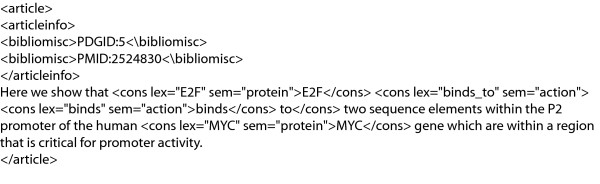
**Refactored corpus: embedded XML format**. Example of the text block from Figure 1 in the refactored embedded XML format.

The refactored PDG corpus, renamed the Protein Interaction Corpus (PICorpus), is publicly available at the BioNLP Sourceforge website [14] in both WordFreak and GENIA-like embedded XML formats. Samples of the two formats are shown in Figures 2 and 3.

### Corpus validation

See Table 1 for a list of validation times for each of the steps. The numbered steps described below correspond to the numbered steps in the Methods section.

**Table 1:** Programming and curation times for each step. Programming times were estimates. Curation times were measured.

Refactor Step	Program	Curation		Total Project	
ID mapping	18 h		10 m		
Finding original sentences	28 h	4 h			
Protein and interaction mapping	32 h	16 h	15 m		
Final formatting	24 h	0 h			

Total time for programming and curation	102 h	20 h	25 m	122 h	25 m

**1. **Very little time was required to validate the PubMed IDs. Only one deprecated MEDLINE ID was not automatically mappable to a PubMed ID. The curator used a portion of the original corpus text string to search for the appropriate article on PubMed and thereby recovered the PubMed ID and abstract manually.

**2. **Verification of the sentence retrieval step took more time and required more effort from the curator. Of the 283 corpus blocks, 33% (96/283) could not be perfectly mapped to a text string in the MEDLINE abstract. Table 2 describes the performance of the automatic sentence extractor. A certain amount of error was introduced because the sentence extraction method compared *single sentences *in the abstract to the original text, which was not necessarily a single sentence. Forty-eight percent (46/96) of these errors were because the evidence text given in the original corpus was in fact more than one consecutive sentence from the abstract. In these cases, the curator manually selected from the retrieved PubMed abstract the multiple sentences indicated in the original corpus. Relaxing the single-sentence requirement could reduce errors in this step by about half. Thirty-nine percent (37/96) had original text that was mined from the title of the publication. This required the curator to search PubMed for the article title, a task that took a quarter of the validation time for this step. In nine of the corpus blocks (9% of the errors) the original corpus text did not span a complete sentence of the abstract, but rather a clause. In those cases the curator kept the full sentence that the automatic system extracted. The choice to change the original content was motivated by the need for context to appear around the protein-protein interactions in the text. Finally, only four mapping errors were introduced by erroneous automatic sentence boundary identification of the abstract text. These were corrected by the curator as well.

**Table 2:** Performance of the automatic sentence extraction step.

Overall performance	Percent	Count
Correct extraction	66%	187/283
Incorrect extraction	33%	96/283
Total	100%	283/283

Type of error	Percent	Count

Too little extracted	48%	46/96
Title text not extracted	39%	37/96
Too much extracted, expanded text selection	9%	9/96
Too much extracted	4%	4/96
Total	100%	96/96

**3. **Validation of the entity mapping step took the longest time – a total of 16 hours and 15 minutes. We made an initial pass at automatic entity location, and spent 9 hours curating the resulting output. This validation pass uncovered a number of systematic errors in our automatic entity tagging. We fixed those errors, reran the entity locator, and then did a second pass. With the improvement in entity location, this second pass took 7 hours and 15 minutes to examine the data and make corrections. Note that some of this time was spent tracking data on the accuracy of the automated step, so the actual validation time is overestimated. The data presented below and in Tables 3 and 4 describe the second entity mapping effort. Of the 283 blocks in the original corpus, 43% (120/283) required manual correction of some kind.

**Table 3:** Results on the automatic entity mapping step

Type of error	Percentage	Count
Text blocks requiring no manual correction	57.6%	163/283
Text blocks requiring at least one boundary correction	22.3%	63/283
Text blocks with at least one unmappable entity	20.1%	57/283

Total	100%	283/283

**Table 4:** Results on named entity mapping: time and required corrections

Curation Step	Number	Time	
a) Manually examine output for validity	n/a	5 h	15 m
b) Fix protein mentions requiring boundary correction	131	1 h	5 m
c) Add protein annotations that were unmappable	42		55 m
d) Remove proteins that were in error in metadata	23		

Total repair time (b + c + d)		2 h	

**Total curation time (a + b + c + d)**		**7 h**	**15 m**

We measured time for three separate tasks in the entity-mapping validation process: manually examining the entire output for correctness, manually fixing cases where the automatic locator found an entity in the text but the boundaries were wrong, and manually fixing cases where the original metadata indicated that an entity existed, but the automatic locator was unable to find it. (This last case had two causes, which we describe below.) The total curation time on the second round was 7 hours and 15 minutes: 5 h 15 m for the manual examination, 55 m for the manual repair of boundaries, and 1 h 5 m for the manual repair of unmapped entities.

Table 4 gives the data for the validation portion of this step. Row (a) shows the time needed to examine the entire automatic output. Row (b) identifies the number of times that the automatic method located an entity within the text, but did not capture its boundaries correctly due to the normalization of entity names in the original corpus. In most cases, this was due to the fact that in the original metadata, entity names like *DP-1 *and *E2F-2 *were normalized to *DP *and *E2F*. In those cases, the automated process located the entities but omitted the hyphenated numerals from the span calculation. The curator adjusted the boundaries manually to include the numerals. We contend that this adjustment corrects an error in the original corpus, but does not change the semantics of the corpus itself. This repair process took 1 h 5 m.

Rows (c) and (d) give the number of times that the original metadata suggested that there was an entity in the text, but the automated step was not able to locate it. There were two causes for this. In 42 cases, it was due to normalization of *unhyphenated *numbers in the metadata, or similar phenomena. For example, the original metadata usually identified *cyclin D1 *as *cyclin D*, and in those cases, the automated step did not find the protein in text. When the automated step missed the annotion, the curator added the entity and its offsets manually. In 23 other cases, the putative entity was not present in the text. For example, in a number of cases, the original corpus metadata suggested that *cyclin destruction *was a token of *cyclin D*. This type of refactoring error reveals errors extant in the original corpus. In such cases, we deleted the erroneous entity from the metadata. Fixing the errors shown in rows (c) and (d) took 55 minutes.

There are 423 interaction types listed in the metadata of the original corpus file. There were 450 interaction tokens found by the entity matcher (some interaction keywords and proteins are mentioned more than once in the evidence text). There were no errors in matching the interaction keywords. There are 696 protein types listed in the metadata of the original corpus file. A total of 935 protein tokens were found in the text by the entity matcher. Of the 696 protein types, 65 were not found by the matcher.

We also noted a number of instances where the interaction type seemed incorrect, or the proteins labelled as taking part in the interaction seemed wrong. In the spirit of keeping the semantics of the corpus constant, we did not modify these. However, we kept notes of these discrepancies and made them available with the corpus download files.

## Conclusion

It is widely accepted in the corpus linguistics community that format is a determinant of corpus usability [5,6,10,22]. As a feasibility study, the work presented here aimed to do two things: 1) answer whether corpus format refactoring is a feasible, tractable problem, and 2) provide insight into the challenges to be faced when refactoring other, bigger corpora.

Regarding feasibility, we found that this corpus could be refactored in about 3 person-weeks' worth of time. While 80% of that time (102 h) was spent programming, many program components of the refactor process can be reused in the next refactoring effort. These components include PubMed ID and MEDLINE abstract retrieval, sentence boundary identification, the protein entity locator, and final format outputting. Components that may need to be written anew for each corpus include a parser for the original corpus file format, entity locators depending on provided metadata (e.g. diseases, drugs, cell types), and the component that loads the annotations into the data structure before final output.

The resulting data from this refactoring project can be used as a gold standard for protein-protein interaction information extraction. This refactored corpus, called the Protein Interaction Corpus (PICorpus), is freely available for download at the BioNLP SourceForge website [14]. A number of enhancements to the corpus are now possible that in its previous format would have been difficult at best. These include, but are not limited to:

• Adding linguistic annotation, e.g. of sentence boundaries and part of speech, which have been contributors to the community acceptance of other corpora such as GENETAG and GENIA.

• Adding annotation of the genes in the text that are *not *involved in the protein-protein interactions, thus making this corpus useful for a new task: entity identification.

• Adding negative examples, making this corpus not just more useful for system evaluation, but amenable to training statistical and machine-learning-based systems.

Using the version control software available on SourceForge, the distribution of iterative feature additions becomes simple.

The *process *of refactoring the PDG brought to light several challenges that are endemic to refactoring projects. The first set of challenges involves how corpus characteristics ease or burden the refactoring process. Specificially,

• Is the original text distributed with the corpus, or does it need to be retrieved?

• Are the spans of entities/relations already available, or do they need to be discovered in text?

• To discover spans, can exact strings be searched for, or do alternate searching techniques have to be used or developed?

Table 5 lists how various biomedical corpora fall into these three categories. The time required to carry out refactoring on a particular corpus is directly related to the answers to these questions. Those corpora that distribute the original text (with no alterations) and the span values for entities and relations will be straightforward to refactor with deterministic programming procedures. Likely little or no curation will be necessary to ensure the refactored version contains the same content.

**Table 5:** Roadmap for refactoring corpora. The list of corpora came from [32] and [33], where there are links to the corpora. Column headings indicate the steps that corpora may need to undergo to be refactored; those corpora that would require that step are noted with a dot. The heading "get original" means the original text needs to be retrieved. "Detect spans" means the corpus is a metadata corpus so spans of entities need to be detected. "Alt. search" means techniques other than exact-match searching must be used.

	get original	detect spans	alt. search
Arabidopsis Thaliana Circadian Rhythms [34]	•		
Bio1 [35]	•		
BioCreative 2004 Task 1A [28]	•		•
BioCreative 2004 Task 1B [36]		•	•
BioCreative 2004 Task 2 [37]		•	•
BioCreative 2006 Task GM [38]			
BioCreative 2006 Task GN [39]			
BioCreative 2006 Task IPS/IMS [40]		•	•
BioCreative 2006 Task ISS [40]		•	
BioInfer [41]			
BioText: Recognizing Abbreviation Defintions [42]			
BioText: Protein-Protein Interaction Data [43]	•		•
BioText: Relations between Disease/Treatment Entities [44]	•		
Brown-Genia Treebank [45]	•		
DepGenia [46]	•		
DIPPPI [47]		•	•
EDGAR [48]	•	•	
GENIA [49, 50]	•		
FetchProt [51]			
Human Gene ID-Serve	•		
IEPA [52]	•	•	
ImmunoTome	•		
iProLink [53]			
Medstract [54, 55]			
MedTag [7]			
OHSUMED [56, 57]	•	•	•
PASBio [58]		•	
PASTA [59]			
PathBinder [60]			
PennBioIE [12]			
PICorpus			
ProSpecTome [61]	•	•	
PDG [9]	•	•	•
Texas [62]	•		•
TREC Genomics 2004 Categorization Task [63]		•	•
TREC Genomics 2005 Categorization Task [64]		•	•
TREC Gemonics 2006 IR Task [65]		•	•
TREC Genomics 2007 IR Task [65]		•	•
Wisconsin [66]	•	•	•
WSD [67]			
Yapex [68, 69]	•		

However, corpora for which the original text has been altered or is not distributed, and for which no annotation span information is available, will require multiple programmatic steps accompanied by validation procedures. Consider, for instance, the validation results in the PDG refactoring process, which required work in all three categories. Take the PDG corpus for example: 33% of the corpus required correction from the sentence matching step, and 43% required correction from the entity matching step. We mentioned earlier that in the original PDG corpus protein entities had been altered in the metadata, an artifact that resulted in significant curation time. In a corpus in which the entities have not been altered, exact string matching techniques may be used. On the other end of the spectrum, in a corpus that provides database IDs of entities, but not text, entity locators may need to rely on natural language processing information extraction techniques. Depending on the degree to which the text was altered in the metadata or annotations, some corpora will require more time than others. Also, programmatic methods that deliver better results will reduce the curation time, and could almost eliminate it.

The original PDG corpus is a metadata corpus. The 20% curation time (20.4 h) for this project came from curating each output block of the corpus. We did a complete curation effort on this project to understand the possible issues. However, a spot-check on some fraction of a refactored corpus is likely to be sufficient. A spot-check will reveal specific refactoring errors, which can then be searched and replaced throughout the entire corpus. At best, the curation time will be constant, regardless of corpus size. At worst, the time needed for curating a refactored corpus will be linear with corpus size. Spot-check curation may result in refactoring inaccuracies in the final output, but with version control software in place, inaccuracies that are found later can easily be fixed and integrated into the publicly available resource.

As mentioned in the Background section, the definition of refactoring is to change the corpus format while preserving the original content. The second set of challenges involves the necessary content changes that we made to the PICorpus despite our goal of not changing any content. We found that in some cases, i.e. when an entity in the metadata could not be found in the text by computer or human, changing the content was inevitable. For the metadata corpora that present this type of challenge, the goal in refactoring is to *minimize *alterations from the original by changing as few annotations as possible.

The third set of challenges involves representational issues. That is, how exactly do we represent the entities and relationships provided in original corpora, especially metadata corpora? Specifically, we are referring to two issues:

• What spans of text should be selected to represent an entity?

• What spans of text should represent the relationship between entities?

The results of step 4 of the curation show that there were some discrepancies between the original and the refactored entity annotations. This is not a novel problem [23-25], and it has been addressed by researchers in a variety of ways. Some researchers have dealt with this problem by developing annotation guidelines that deal explicitly with entity spans [26,27]. Others have accounted for the variability by developing metrics that measure span boundary matches [25], or by recognizing possible variants as correct, a tack taken by the BioCreative shared task evaluators [28].

The second representational issue involves how to encode the relationships between entities. For instance, in the refactored PICorpus described here, the relationship between the two interacting proteins is represented by annotating the span of the interation keyword in text with the annotation "action." An alternative would have been to select the span of text from the first keyword or protein involved in the interaction through the last. Yet another alternative would be to provide dependency-style information within the annotation that links proteins through the interaction keywords to their interacting counterparts, a style used by PropBank and NomBank [29,30].

A final refactoring challenge was illuminated by curator feedback: the curators found the presentation of the data difficult to read during the validation process. Curators were given plain text files that displayed the original corpus text and the span values for the particular entities identified in text that they were to be checking, i.e. PMID, sentences, or proteins/interaction types. Besides direct curator feedback, this difficulty is also evidenced in Table 4 by the disparity between the time reported to examine the corpus for error (5 h 15 m) and time to make repairs on the data (2 h). In future refactoring efforts, we will consider loading data at each step into an annotation tool, such as Knowtator [31], to ease the burden on the curators.

How a corpus gets annotated is often driven by what specific task the annotators expect to use the corpus for, and different tasks will dictate a corpus be annotated differently. However, with automatic refactoring methods in place, changing from one style of annotation to another need not be an intractable process.

## Authors' contributions

KBC conceived the original idea. LH and KBC supervised all steps of the work. HLJ programmed the parsing and mapping steps. WAB programmed the output step. HLJ, MK, and KBC each curated portions of the data. HLJ and KBC wrote the manuscript. MK, WAB, and LH edited and approved the manuscript.
